# An Adhesive/Anti‐Adhesive Janus Tissue Patch for Efficient Closure of Bleeding Tissue with Inhibited Postoperative Adhesion

**DOI:** 10.1002/advs.202301427

**Published:** 2023-05-12

**Authors:** Wan Peng, Cheng Liu, Youjin Lai, Yanting Wang, Pingsheng Liu, Jian Shen

**Affiliations:** ^1^ Jiangsu Collaborative Innovation Center of Biomedical Functional Materials Jiangsu Key Laboratory of Bio‐functional Materials School of Chemistry and Materials Science Nanjing Normal University Nanjing 210023 P. R. China; ^2^ The Affiliated Drum Tower Hospital of Nanjing University Medical School Nanjing 210093 P. R. China; ^3^ Jiangsu Engineering Research Center of Interfacial Chemistry Nanjing University Nanjing 210093 P. R. China

**Keywords:** anti‐postoperative adhesion, hemostasis, injured bleeding tissues, *Janus* tissue patch, wet tissue adhesive, °C

## Abstract

Most of the current bioadhesives cannot perform well on bleeding tissues while postoperative adhesion is a general but serious clinical issue. Here, a three‐layer biodegradable *Janus* tissue patch (J‐TP) that is able to simultaneously enable efficient closure of bleeding wounds with significantly promoted clotting ability and suppressed postoperative adhesion of tissues is reported. A dry adhesive hydrogel bottom layer of the J‐TP can form rapid (within 15 s) and strong (tensile strength up to 98 kPa) adhesion to bleeding/wet tissues with high bursting pressure (about 312.5 mmHg on a sealed porcine skin) through hydrogen binding and covalent conjugation between the carboxyl & *N*‐hydroxy succinimide (NHS) groups of hydrogel and the primary amine groups of tissues, while the phosphonic motifs can significantly reduce blood loss (by 81% on a rat bleeding liver model) of bleeding wounds. A thin polylactic acid (PLA) middle layer can improve the tensile strength (by 132%) of the J‐TP in wet conditions while the grafted zwitterionic polymers can effectively prevent postoperative tissue adhesion and inflammatory reaction. This J‐TP may be a promising tissue patch to assist the clinical treatment of injured bleeding tissues with inhibited postoperative adhesion.

## Graphical abstract

A novel *Janus* tissue patch where one side can form quick and robust adhesion to diverse wet/bleeding tissues with promoted coagulation properties, while the other side can inhibit cell/tissue adhesion due to the intrinsic antifouling ability. This *Janus* tissue patch can synergistically address the weak performance on bleeding tissues and the severe postoperative adhesion that is found on most of the current bioadhesives.

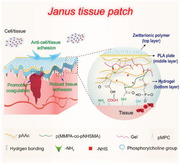

## 1 Introduction

In military wars and civilian accidents, tissue trauma and associated acute bleeding are the major causes of mortalities, leading to more than 2 million annual deaths globally,^[^ ^1^ ^]^ while up to 40% of these cases are caused by hemorrhage.^[^ ^2^ ^]^ Unlike minor bleeding that can be prevented by innate hemostatic mechanisms, massive bleeding requires instant hemostatic interventions to prevent excessive blood loss, avoiding the occurrence of hypovolemia, coagulopathy, tissue death, and hemorrhage shock.^[^ ^3^, ^4^ ^]^ Current clinical treatments for bleeding injuries usually contain two steps: stop bleeding and then suture the wound,^[^ ^5^ ^]^ where cotton gauze is the most widely used hemostatic material for traumatic bleeding control via concentrating blood's hemostatic components to stop bleeding.^[^ ^3^ ^]^ However, this process may result in excessive blood loss because a large volume of blood is absorbed by gauze before the complete stop of bleeding. Furthermore, time‐consuming, complicated operations and tissue damage are typical disadvantages of suturing that greatly limit the treatment efficiency of bleeding tissue and reduce patients’ survival.

In recent years, the adhesive sealant is an attractive alternative to suturing^[^ ^6^ ^]^ because of its easy & rapid operation and good sealing performance on bleeding/wet tissues.^[^ ^7^ ^]^ As estimated, the surgical adhesive market in the US is about $1.8 billion with an annual growth rate of 9.7%.^[^ ^8^ ^]^ Commercially available tissue adhesives, such as polyethylene glycol‐based hydrogel sealants,^[^ ^9^ ^]^ cyanoacrylates,^[^ ^10^ ^]^ fibrin glue,^[^ ^11^ ^]^ and glutaraldehyde cross‐linked albumin,^[^ ^12^ ^]^ have been widely applied in clinic laparoscopic, minimally invasive and microsurgical procedures.^[^ ^13^, ^14^ ^]^ However, these commercial tissue adhesives normally cannot work well on bleeding tissues. For example, though fibrin glue can enable bleeding control through coagulation‐dependent hemostasis, the adhesion to tissues is very weak, thus it could be washed away by blood flushing. Cyanoacrylates can stop bleeding because of their rapid polymerization/gelation on the wounding site, but their toxicity and bad biodegradability are non‐negligible disadvantages for tissue healing. Therefore, the development of a new bioadhesive with excellent biocompatibility, biodegradability, and robust adhesion and sealing of injured bleeding tissues is urgently required.

During the past decade, many hydrogel‐based tissue adhesives have been developed mainly relying on three mechanisms: 1) diffusion of polymeric chains through interfacial water to form interpenetrated networks with tissues,^[^ ^15^, ^16^ ^]^ 2) removal of interfacial water to form tight bonding with tissues,^[^ ^16^, ^17^, ^18^ ^]^ 3) covalent conjugation to wet tissues.^[^ ^19^ ^]^ Usually, bulk hydrogel^[^ ^15^, ^20^ ^]^ and injectable hydrogel^[^ ^21^, ^22^, ^23^ ^]^ adhesives mainly adopt the first mechanism to achieve hemostasis. Bulk hydrogel adhesives are easy to use, but time‐consuming to form bonding with bleeding tissues due to low diffusion efficiency to tissue network.^[^ ^24^ ^]^ Injectable hydrogel adhesives have morphologically adaptability to difform wounds, and can strongly adhere to tissues owing to the rapid diffusion of injectable hydrogel precursors into surrounding tissues before gelation.^[^ ^23^, ^25^ ^]^ However, these hydrogel precursors are easy to be diluted by blood/body fluid and usually need auxiliary UV (ultraviolet) to achieve gelation. Recently, tissue adhesives that employ the water‐removal adhesion mechanism have attracted great attention. Depending on absorbing/repelling water from the tissue surface, these bioadhesives can pass through the interfacial water and permeate into the tissue network quickly (within 10 s^[^ ^16^ ^]^), resulting in fast and robust adhesion with wet tissues.^[^ ^26^ ^]^ In addition, considering that covalent bonds are more stable than non‐covalent bonds under wet conditions, many bioadhesives with covalent bonds (such as Schiff base bond,^[^ ^22^ ^]^ active ester bond,^[^ ^16^, ^19^ ^]^ and isocyanate bond^[^ ^5^ ^]^) to tissues have been shown enhanced adhesion strength and stability.

So far, numerous tissue adhesives with good tissue adhesion ability have been developed following these three adhesion mechanisms. However, most of these bioadhesives are designed as double‐side adhesion, which inevitably lead to undesired postsurgical tissue adhesion and scar tissue formation due to their indiscriminate adhesion to surrounding tissues/organs and adhesive‐induced inflammatory response. Consequently, readmission and re‐operation to avoid the occurrence of chronic pelvic pain, intestinal obstruction, and infertility are required.^[^ ^27^, ^28^ ^]^

Hence, in the current study, we designed a novel adhesive/anti‐adhesive *Janus* tissue patch in that one side is able to strongly stick to bleeding tissues while the other side is able to prevent postoperative cell/tissue adhesion efficiently. The J‐TP combines a sticky dry hydrogel bottom layer based on the water‐removal mechanism with active ester (NHS) pendants and coagulating phosphonic acid polymers (pMMPA) engineered, a thin PLA plate middle layer and a non‐adhesive antifouling top layer. The chemical structures and compositions of MMPA polymers were characterized by nuclear magnetic resonance (NMR). The chemical compositions of J‐TP were characterized by X‐ray photoelectron spectroscopy (XPS). The mechanical property of the tissue patch with/without a thin PLA plate was tested. The cytocompatibility, antifouling property, adhesive ability to various wet tissues (skin, liver, stomach, and heart), as well as hemostatic ability of J‐TP, were systematically investigated in vitro and ex vivo. The biodegradability of J‐TP was investigated both in vitro and in vivo. Furthermore, the anti‐tissue adhesion property of the top layer of J‐TP and the sealing performance of J‐TP toward bleeding rat liver were evaluated in vivo.

## 2 Results and Discussion

### 2.1 Design, Preparation, and Characterization of J‐TP

**Figure 1:**
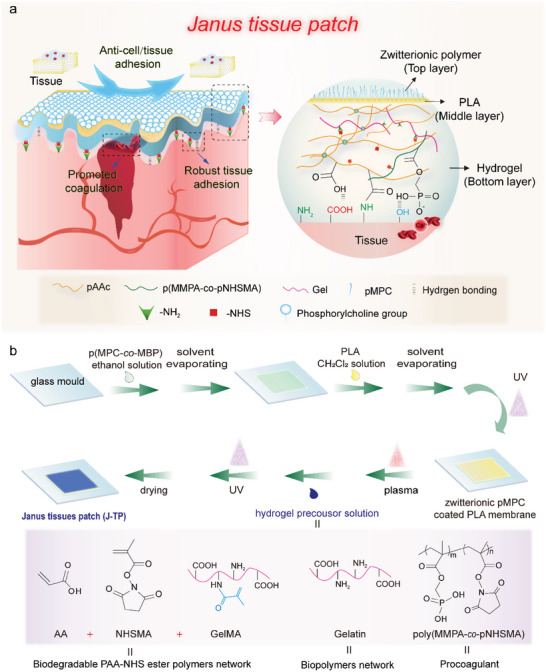
Design and preparation of J‐TP. a) Schematic illustration of the design and structure of J‐TP. b) Schematic illustration of the preparation process of J‐TP.

In order to enable an efficient closure of bleeding wounds with promoted clotting and to simultaneously inhibit the postoperative tissue adhesion, a novel adhesive/anti‐adhesive *Janus* tissue patch (one side is able to strongly stick to bleeding tissues while the other side is able to prevent postoperative cell/tissue adhesion) was designed (**Figure** **1**a). The dry hydrogel adhesive & coagulating bottom layer is mainly composed of two crosslinked networks: poly(acrylic acid)‐*co*‐poly(methacrylic acid N‐hydroxysuccinimide ester) (p(AA‐*co*‐NHSMA)) polymers network crosslinked by biodegradable GelMA and gelatin biopolymers network crosslinked by hydrogen bonds. Since the phosphonic polymers can initiate the inherent coagulation pathway to promote clotting,^[^ ^29^, ^30^ ^]^ a MMPA‐based polymer (p(MMPA‐*co*‐NHSMA)), as a potential coagulant, was incorporated into the bottom hydrogel layer through hydrogen bonds and covalent bonds between NHS ester and primary amino groups of gelatin. The abundant negatively charged carboxylic acid and phosphonic acid groups in the bottom layer accelerate the hydration of dry hydrogel to dry the wet tissue surface. Meanwhile, the carboxylic acid groups form hydrogen bonds and electrostatic interaction with the tissue surface to achieve primary adhesion, and the phosphonic acid groups activate the contact pathway to promote clotting. In a few minutes, the NHS ester pendants in p(AA‐*co*‐NHSMA) polymers react with primary amine groups on the tissue surface to form stable adhesion.^[^ ^5^ ^]^ Furthermore, the usage of a biodegradable crosslink agent (GelMA) and high content of gelatin (10%) endow the hydrogel layer with good biodegradability and biocompatibility. The middle layer is a thin PLA plate as the skeleton of the patch, while the third layer is a zwitterionic polymer coating that was grafted on the PLA plate as the antifouling top layer of J‐TP to combat the cell/tissue adhesion.

**Figure 2:**
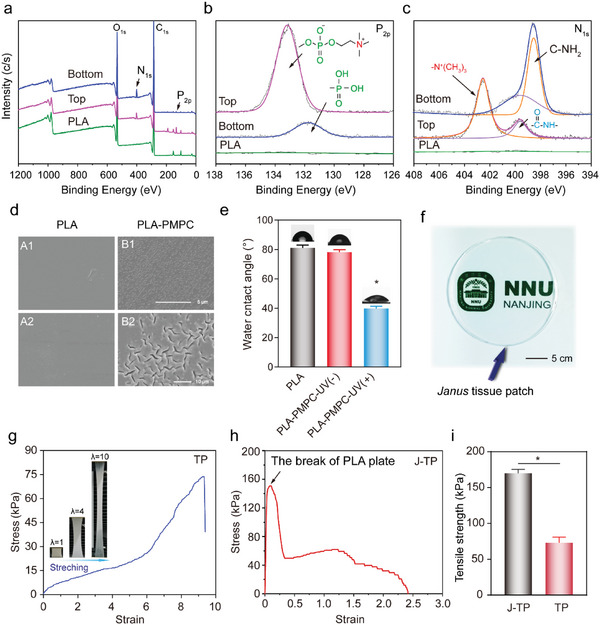
Characterization of the J‐TP. a) XPS survey scan and b,c) high‐resolution scan spectra of the adhesive/antifouling layers of J‐TP. d) SEM images of (A1&A2) PLA, (B1&B2) PLA‐PMPC film surfaces morphology. Magnification: ×500 (A1, B1), ×2000 (A2, B2). e) Water contact angle on ultrasonic cleaning PLA, PLA‐PMPC‐UV(−), PLA‐PMPC‐UV(+) film surface. UV(−)/UV(+) represents the presence/absence of UV irradiation after PLA deposition on p(MPC‐co‐MBP) copolymers, respectively. f) Exhibition of the transparent J‐TP. Stretching images and tensile strength versus stretch curve for g) TP and h) J‐TP in swollen state. i) Tensile strength of J‐TP and TP. Data are shown as mean ± SEM (*n* = 3). Pairwise comparisons of different groups are statistically significant (*, *p* < 0.05) as determined by one‐way ANOVA multiple comparisons or Student's *t*‐test.

To prepare the *Janus* tissue patch, a layer‐by‐layer construction process (as shown in Figure 1b) was performed. To prepare the antifouling layer, the photoreactive copolymers p(MPC‐*co*‐MBP, 80:20) that bear a zwitterionic phosphorylcholine group were deposited on a glass mold, followed by the deposition of PLA. The p(MPC‐*co*‐MBP) copolymers could graft onto PLA plate under UV irradiation due to the photoreactive benzophenone group in the p(MPC‐*co*‐MBP) copolymers which can abstract aliphatic hydrogens to form grafting. To verify the presence of poly(MPC‐*co*‐MBP) copolymers on the PLA film, the surface elemental composition, wettability, and morphology were characterized by XPS, WCA, and SEM, respectively (**Figure** **2**a–e). As compared to that of pristine PLA, the XPS spectrum of PLA‐PMPC film showed strong characteristic signals of phosphorus (at a binding energy of ≈133 eV, corresponding to phosphorylcholine moiety in MPC) and nitrogen (≈400 eV, corresponding to the phosphorylcholine moiety in MPC and the amide group in MBP) (Figure 2a). Further N_1S_ high‐resolution scan spectra showed that two nitrogen species were detected on PLA‐PMPC surface: ‐N(CH_3_)_3_^+^ species (about 76.6%) at the binding energy of ≈402.5 eV and O=C–NH‐ species (about 23.4%) at the binding energy of ≈399.5 eV (Figure 2b,c). In addition, no characteristic signals of phosphorus and nitrogen have been detected on the PLA‐PMPC‐UV(−) surface (Figure S2, Supporting Information), further indicating that the chemical coating of p(MPC‐co‐MBP) copolymers on the PLA surface. These data clearly indicated the successful construction of zwitterionic polyMPC coating on the PLA plate. When the p(MPC‐*co*‐MBP) polymers have been grafted onto the PLA plate, it exhibits rougher surface morphology as compared to the PLA surface (Figure 2d), similar to that observed on the surface of silicone rubber.^[^ ^31^ ^]^ In addition, the surface wettability of the PLA plate was significantly improved due to the presence of highly hydrophilic MPC polymers on the surface (Figure 2e).

To prepare the tissue adhesive/hemostasis hydrogel layer of J‐TP, a hydrogel precursor solution (as shown in Figure 1b) was added to the plasma‐treated PLA plate and was gelled under UV light. The hydrogel was crosslinked by GelMA (Figure S1, Supporting Information), while the pMN copolymers can be grafted onto the hydrogel network based on the NHS/NH_2_ chemistry (Figure 1a). XPS results revealed the presence of a characteristic signal of P_2p_ at a binding energy of 132 eV (Figure 2a,b, attributed to the phosphonic group in pMN copolymers), indicating the successful incorporation of phosphonic polymers in the hydrogel with good transparency (Figure 2f). Owing to the incorporation of a thin PLA plate, the tensile strength of the hydrogel tissue patch can be doubled (169 kPa of J‐TP vs 73 kPa of TP, Figure 2g–i), offering good stability of the tissue patch once adhered to wet tissues and good mechanical match to biological tissues.^[^ ^5^ ^]^

### 2.2 Adhesion Performance of J‐TP

Rapid and robust adhesion capability in a wet environment is essential for bioadhesives during wound healing, as tissue damage is often accompanied by bleeding and exudation of body fluid. Compared with wet hydrogels, dry hydrogels allow closer contact with tissues based on the water removal mechanism, exhibiting outstanding advantages in achieving fast adhesion to tissues.^[^ ^16^ ^]^

**Figure 3:**
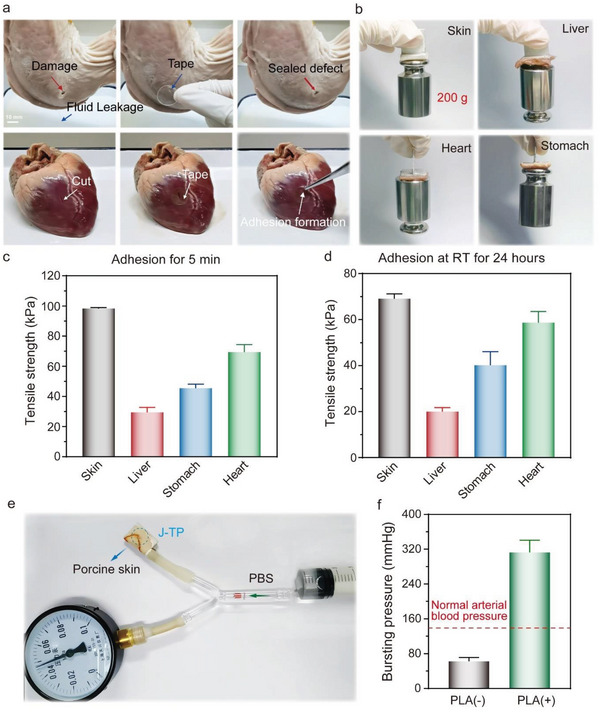
Adhesive performance of the J‐TP. a) The adhesive performance of the bottom surface of J‐TP to wet porcine stomach with fluid exudation and heart within 15 s. b) The long‐term (24 h) adhesive performance of J‐TP to porcine skin, liver, stomach, and heart; All the adhered model can lift a weight of 200 g. c) Short‐term (5 min) tensile strength between various tissues adhered by the J‐TP (*n* = 3). d) Short‐term (24 h) tensile strength between various tissues adhered by the J‐TP (*n* = 3). e) The digital photo of the model for measuring the bursting pressure. f) Quantitative analysis of the bursting pressure; Data are shown as mean ± SEM (*n* = 3). Pairwise comparisons are significantly different (*, *p* < 0.05) as determined by Student's *t*‐test.

To evaluate the adhesion performance of J‐TP, the adhesion of J‐TP to diverse fresh animal organs (e.g., porcine skin, liver, stomach, and heart) was tested. The negatively charged adhesive layer of J‐TP could absorb water quickly to form a thin adhesive hydrogel and then adhere to the tissue surface depending on the abundant hydrogen bonding between the carboxylic/phosphonic acid groups in adhesive hydrogel and the hydroxyl, carboxyl, and amino groups on tissues surface. In addition, the NHS groups in the hydrogel could react with the amino groups on the tissues’ surface in minutes,^[^ ^16^ ^]^ forming robust and durable adhesion through covalent conjugation in a wet environment (Figure 1a). As shown in **Figure** **3**a and Figure S3, Supporting Information, the J‐TP enabled stable adhesion within 15 s with wet tissues at 37 ° C (close to the curing time (≈10 s) of commercial Fibrin glue), resulting in a quick sealing of tissue wounds with fluid exudation (Movies S1 and S2, Supporting Information). Even after 24 h in a wet environment, the J‐TP could still bear a weight of 200 g (Figure 3b), indicating a strong/stable tissue adhesion capability. Further quantitative tensile strength (Figure S4a, Supporting Information) tests showed that the adhesion strength between J‐TP and porcine skin, liver, stomach, and heart were 98.3, 29.4, 45.4, and 69.4 kPa within 5 min, respectively. The short‐term adhesion strength values of J‐TP to biological tissues are comparable to the reported tough hydrogel bioadhesive study^[^ ^18^ ^]^ or ultra‐strong bio‐glue.^[^ ^15^ ^]^ And it has largely remained even after 24 h (69.1, 20.0, 40.2, and 58.7 kPa, respectively) (Figure 3c,d and Figure S4, Supporting Information), suggesting a robust adhesion capability that is superior to many commercial bioadhesives.^[^ ^16^, ^18^ ^]^ It should be noted that unlike the trends observed on other tissues, the adhesion strength of J‐TP to porcine skins performed an obvious decrease from 5 min to 24 h, which may be related to more grease exiting in porcine skin than other tissues. Moreover, a facile model was designed to measure the burst pressure of J‐TP on porcine skins (Figure 3e). As shown in Figure 3f and Movies S3 and S4, Supporting Information, the bursting pressure of the J‐TP was 312.5 mmHg, much higher than that of the normal arterial blood pressure (~140 mmHg). Notably, the thin PLA plate increased the bursting pressure by five folders of the TP (312.5 vs 62.5 mmHg), which could satisfy the requirements of bleeding wound sealing.

### 2.3 Antifouling Performance, Biocompatibility, and Biodegradability of J‐TP

Postoperative adhesion to surrounding tissues is a general but serious clinical issue of biomedical implants and devices. Most existing bioadhesives are unable to avoid postoperative adhesion due to their intrinsic double‐sided adhesion property, thereby requiring adhesion lysis operation and readmission.^[^ ^28^ ^]^ Zwitterionic polymers are a type of synthetic material with excellent antifouling ability due to their unique structure (equal amounts of cationic and anionic groups exist in the polymer chains).^[^ ^32^ ^]^ It can form a tight hydration shell around through hydrogen bonding and electrostatic interaction, and be able to repel protein adsorption, bacteria, and cell adhesion associated with infection and postoperative adhesion formation.^[^ ^27^, ^31^, ^33^ ^]^

**Figure 4:**
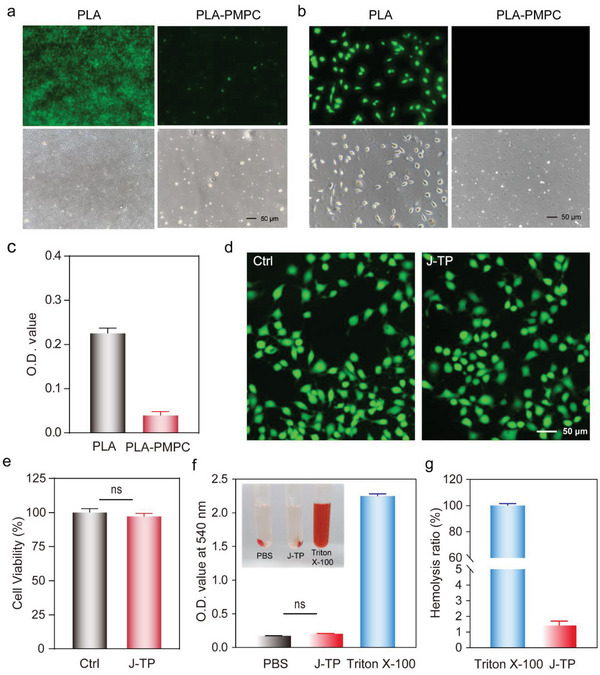
In vitro antifouling performance and biocompatibility of J‐TP. a) Fluorescence microscopy images of L929 cells attached on PLA and PLA‐PMPC surfaces after 24 h incubation. b) Quantitative analysis of L929 cells attached on PLA and PLA‐PMPC surfaces as determined by the CCK‐8 cell viability assay. c) Fluorescence microscopy images of *S. aureus* adhered on PLA and PLA‐PMPC surfaces after 12 h incubation. d) Fluorescence microscope images of live/dead L929 cells stained by calcein/PI cell viability assay kit. e) Cell viability assay of L929 cells on different substrates after 24 h of incubation. Pairwise comparisons are not significantly different (ns, *p* > 0.05) as determined by Student's *t*‐test. f) O.D. value of supernatant of PBS, J‐TP, and Triton ×100 incubated with rat red cells at 37 °C for 1 h. Statistical significance was determined by one‐way ANOVA multiple comparisons. g) Hemolysis ratio of J‐TP. Statistical significance was determined by Student's *t*‐test. Data are shown as mean ± SEM (*n* = 3).

To evaluate the antifouling performance of the top layer of J‐TP, the capabilities of J‐TP in resisting cellular and bacterial adhesion were investigated in vitro. As tested, numerous L929 cells were observed on the PLA surface while no obvious cell attachment was observed on the PLA‐PMPC surface (**Figure** **4**a). Quantitative data further verified the significantly suppressed cell attachment on the PLA‐PMPC surface as compared to that on the PLA surface (Figure 4b). Similarly, *Staphylococcus aureus* that attached to the PLA plate has already developed into dense biofilm after incubation (12 h) as indicated by the fluorescence image (Figure 4c), while only minimal *S. aureus* colonies were observed on the PLA‐PMPC surface (Figure 4c). Further cell viability assay revealed that the pMPC does not compromise the cytocompatibility of the tissue patch (Figure 4d,e). And hemolytic activity assay revealed that J‐TP in contact with red blood cells showed a mean hemolysis ratio (1.41%) of less than 1.5%, which was below the ASTM standard of 5% (F756‐2008),^[^ ^34^ ^]^ indicating the good hemocompatibility of J‐TP (Figure 4f,g).

**Figure 5:**
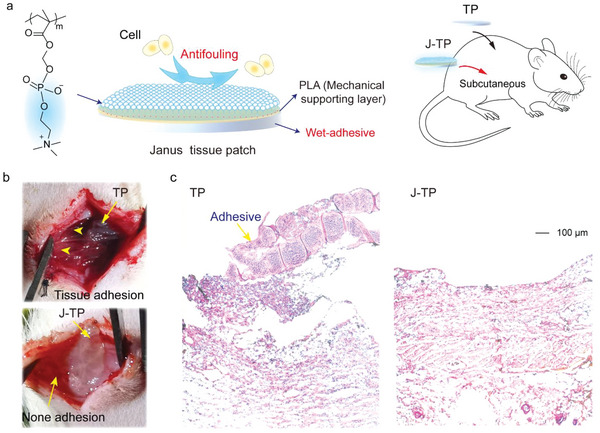
In vivo antifouling performance. a) Schematic illustration of the J‐TP structure and the establishment of rat subcutaneous implantation model. b) The gross images of adhesives implanted in rat after a week. c) The H&E staining images of tissues around the implanted adhesives. TP represents the tissue patch without the PLA layer and zwitterionic layer.

To further confirm the J‐TP's performance in preventing tissue adhesion, a subcutaneous implantation model on rats^[^ ^31^ ^]^ was performed (**Figure** **5**a). After 7 days of implantation, severe patch/tissue adhesion was observed in the TP group (Figure 5b). Further H&E staining images not only verified the severe postoperative tissue adhesion but also indicated obvious inflammatory responses to the TP implant for massive inflammatory cells presented at the adhesive/tissues interface (Figure 5c). In vast contrast, no obvious tissue adhesion was observed between the top surface of the J‐TP and the tissues (Figure 5b). Only minimal inflammation occurred around the J‐TP (Figure 5c). This could be mainly attributed to the presence of a zwitterionic layer on the J‐TP surface, which can resist non‐specific protein absorption^[^ ^35^ ^]^ and thereby avoid recognition by macrophages.^[^ ^36^ ^]^ All these data suggest that the zwitterionic polymer coating on the J‐TP surface could endow good anti‐postoperative tissue adhesion ability to the J‐TP.

### 2.4 Ex Vivo Hemostatic Performance of J‐TP

Hemostatic agents or dressings are often used to stop bleeding. Usually, they work through three major mechanisms:^[^ ^37^ ^]^ 1) concentrating blood's hemostatic components by absorbing water from blood to accelerate coagulation; 2) activating the blood coagulation cascade; 3) providing a physical barrier to blood flow. When used for bleeding wounds, the dry tissue patch can absorb water from blood to concentrate the blood components in contact with the J‐TP adhesive layer. A previous study has shown that polyP (highly anionic linear polymers of inorganic phosphate) serving as a coagulant can be added to wound dressings to accelerate coagulation.^[^ ^38^ ^]^ Considering that the MMPA polymer has similar structure to polyP, combined with its good potency to accelerate platelet adhesion and activation,^[^ ^39^ ^]^ we hypothesized that it could promote the clotting ability of J‐TP.

**Figure 6:**
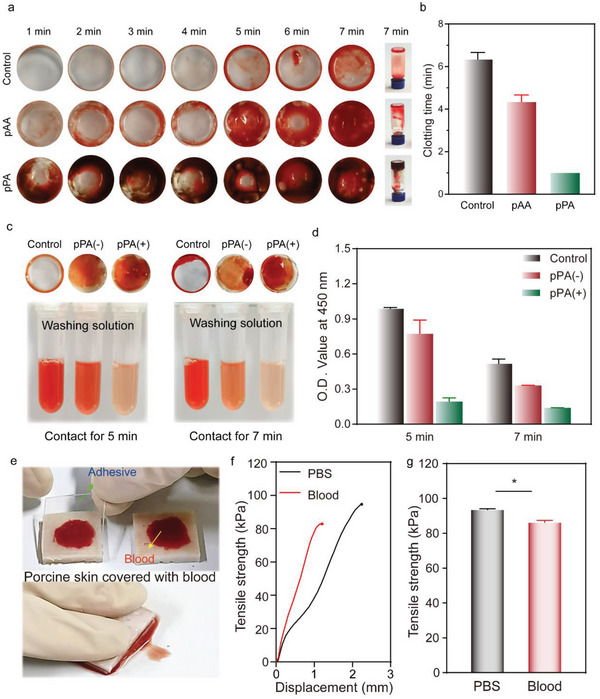
Ex vivo hemostatic performance of J‐TP. a) Blood clotting kinetics of the MMPA polymers (pPA). b) A quantitative analysis of blood clotting time. c) At 5 and 7 min, the blood clotting images after washing and the recorded images of the washing solution. pPA(−) and pPA(+) represent the J‐TP without and with pPA polymers, respectively. d) A quantitative analysis of the optical density of the washing solution. e) Images of the adhesion process between J‐TP and blood‐contaminated porcine skin. f) Tensile strength versus displacement curves for tensile tests of blood‐covered porcine skins adhered by the J‐TP for 5 min. g) A quantitative analysis of the adhesion strength between J‐TP and blood‐covered porcine skin (PBS‐wetted skin as the control group). Data are shown as mean ± SEM (*n* = 3). Pairwise comparisons of different groups are statistically significant (*, *p* < 0.05).

To test the hemostatic performances of J‐TP, the ex vivo blood clotting time of pMMPA polymers (pPA) and anionic pAA (positive control) was investigated according to the published protocol.^[^ ^40^ ^]^ As shown in **Figure** **6**a,b, the clotting time of the negative control group was about 7 min. It was reduced to 5 min with the introduction of pAA in the mixture. In vast contrast, massive blood clots in dark red color were formed within 1 min in the presence of pPA in the mixture. And a bulk blood clot was formed on the bottom of the glass vial after 7 min, indicating that MMPA polymers could be an excellent procoagulant. This could be attributed to its highly anionic polymer chains, which could integrate with FXII and induce the activation of FXII to initiate the contact coagulation pathway.^[^ ^29^ ^]^ Moreover, the incorporation of pMMPA polymers in J‐TP (pPA+ group) (covalently grafted onto hydrogel network through the NHS/NH_2_ chemistry) could also significantly promote coagulation as indicated by less red cells remained in the washing solution as compared to that of J‐TP without pMMPA polymers (pPA‐ group) (Figure 6c,d).

In addition, whether the bleeding affects the adhesive performance of J‐TP was further tested. With the proper amount of fresh blood dropped on porcine skin, the J‐TP was quickly put on top of the blood and pressed for 5 min. As tested, the tensile strength of J‐TP that covered the blood/ porcine skin was 85.9 kPa, only 8% decrease as compared to that of the control group (Figure 6e–g).

### 2.5 In Vivo Hemostasis and Wound Healing Performance

**Figure 7:**
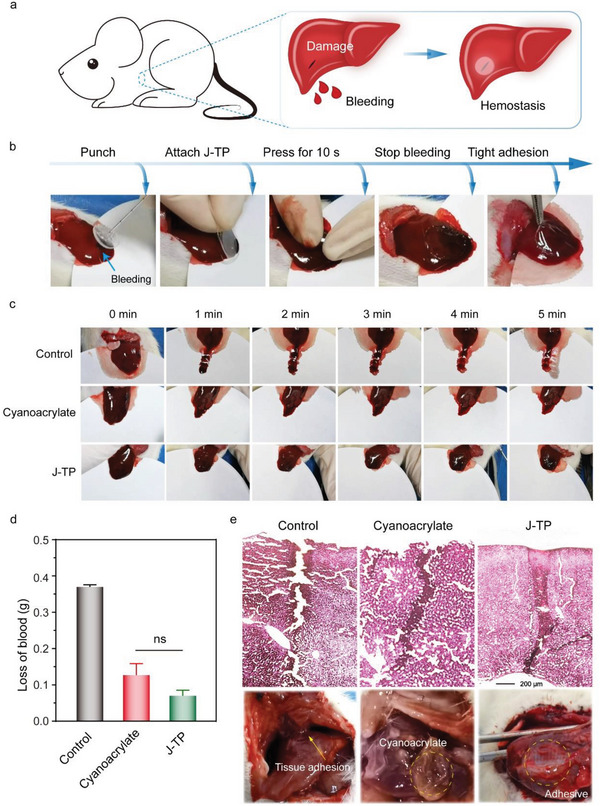
In vivo hemostatic sealing of a rat liver injury. a) Schematic illustration of the establishment of the liver bleeding model. b) Images of the hemostatic procedure of the J‐TP. c) Time‐course bleeding images of the liver. d) Quantitative analysis of blood loss. Data are shown as mean ± SEM (*n* = 3). Statistical significance was determined by one‐way ANOVA multiple comparisons. Pairwise comparisons of different groups are statistically significant unless denoted as “ns” (not significant). e) H&E staining images and gross observation of the wound on day 5.

To further evaluate the hemostatic adhesive of J‐TP, an in vivo bleeding model on rat liver was performed (**Figure** **7**a). The blood loss and the repairing of injured liver tissue treated by J‐TP and the commercially used cyanoacrylate glue were compared, while the injured liver without any treatment was set as the negative control. Though the bleeding in the rat liver can stop at about 60 s in the control group due to the body's natural coagulation pathway (Figure 7c and Movie S5, Supporting Information), the total loss of blood during the process is about 310 mg (Figure 7d). As a clinic used glue, cyanoacrylate is able to quickly stop the bleeding and seal the wound due to the quick polymerization and gelation on the injured site of the wound (Movie S6, Supporting Information). Therefore, it enabled a significantly decreased loss of blood (127 mg, Figure 7d). Interestingly, the J‐TP could adhere to the bleeding surgical site of the liver rapidly and firmly even only after 10 s of pressing (Figure 7b), the amount of blood loss in the J‐TP group was 70 mg, reducing by 81% as compared with that of the control group (superior to the cyanoacrylate group, which was able to reduce blood loss by 66%, and close to decreased blood loss (79%) of the reported visible light‐induced blood‐resistant hemostatic adhesive^[^ ^40^ ^]^).

To evaluate the J‐TP‐assisted healing of the liver injury, liver tissues were sectioned for histological analysis on day 5 post‐operation. For the untreated control group, an obvious gap still presented on the liver after five days, accompanied by an undesirable adhesion to the peritoneum (Figure 7e). Comparatively, the cyanoacrylate‐treated group showed a better re‐connection of the separated wound tissues. However, the gelled cyanoacrylate glue is non‐biodegradable and cannot be removed from the healed liver tissue without operations. Remarkably, no undesirable adhesion to the peritoneum was observed on the J‐TP‐sealed liver wound. More importantly, the liver wound was completely closed by the newly formed liver tissues.

Recently, several bleeding tissue adhesives have been developed to achieve rapid sealing of bleeding tissues.^[^ ^14^, ^41^, ^42^, ^43^ ^]^ However, these existing bioadhesives either need physical assistance (e.g., UV irradiation)^[^ ^14^, ^41^ ^]^ or long pressure time (in minutes)^[^ ^43^ ^]^ to achieve the sealing, which largely compromises the efficiency in clinic applications, while the postoperative adhesion is a general but serious clinical issue. The above data strongly suggested that J‐TP could be a potential adhesive for rapid hemostatic control with promoted wound healing for clinic applications.

### 2.6 Biodegradable Performance of J‐TP

Proper degradability is necessary for bioadhesives during tissue healing. Cyanoacrylate is one of the commercial bioadhesives that has been widely used in clinics. Though it can enable quick gelation and closure of tissue/organ wounds, its intrinsic non‐biodegradability largely hampers its application. In the current study, as biodegradable biomacromolecules (gelatin), polymers (PLA), and crosslinkers (gelatin methacrylate) were used in the preparation of the novel tissue patch, the biodegradation property of the J‐TP have been investigated both in vitro and in vivo.

**Figure 8:**
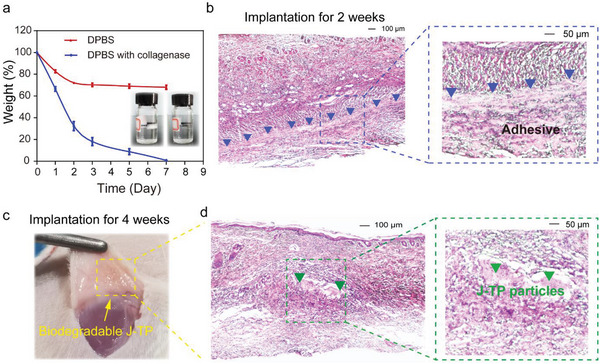
Biodegradability of J‐TP. a) In vitro biodegradation of the J‐TP in DPBS with/without collagenase. b) Representative H&E staining images of tissues around the adhesives after 2 weeks of subcutaneous implantation on a rat. Representative c) in situ image and d) H&E staining images of tissues around the adhesives after 4 weeks of subcutaneous implantation on a rat.

In vitro degradation results clearly showed that the J‐TP can be completely degraded by endogenous enzymes in PBS within 7 days (**Figure** **8**a), indicating a good biodegradability of J‐TP that is suitable for potential clinical applications. However, an ideal tissue adhesive is expected to be degraded at a suitable rate that matches the wound healing process.^[^ ^5^ ^]^ Bioadhesives wtih rapid degradation rates may increase the risk of blood or other body fluid leakage, while a too‐slow degradation rate may lead to prolonged inflammation and tissue fibrosis. Therefore, the in vivo biodegradability of J‐TP based on a subcutaneous implantation model on rats for up to 4 weeks was further performed. Interestingly, after 2 weeks of subcutaneous implantation, strong adhesion between the skin tissue and the J‐TP was clearly observed as indicated by the linear tissue/adhesive interface (denoted by blue triangles in Figure 8b). After 4 weeks of subcutaneous implantation, only a small amount of J‐TP trace remained at the surgical site (Figure 8c). Further microscopic H&E histological images also showed that only a few of the hydrogel pieces were embedded by tissues (denoted by green triangles in Figure 8d), indicating the majority of the J‐TP was degraded at the 4‐week period, suggesting that the moderate in vivo degradation rate of J‐TP matches well with the wound healing process.

## 3 Conclusion

In summary, we report a novel biodegradable *Janus* tissue patch for the hemostatic sealing of bleeding tissues. The bottom layer of J‐TP synergistically combines adhesive (PAA & NHS) and thromboplastic (pMMPA) hydrogel network that is capable of enabling rapid, robust, and hemostatic sealing of bleeding tissues. The thin PLA plate middle layer can prevent the quick swelling of the hydrogel layer under wet conditions, thus providing good overall mechanical properties under physiological/bleeding conditions. The zwitterionic pMPC top layer can efficiently inhibit the cell/tissue adhesion on the patch after the sealing of the bleeding tissue. Therefore, the rapid hemostatic sealing capability, combined with the excellent antifouling performance of the *Janus* patch could simultaneously address the weak adhesive performance on bleeding tissues and the general but serious postoperative adhesion issues of the existing bioadhesives. Moreover, the *Janus* tissue patch can strongly stick to the tissue by 2 weeks and can be near completely degraded by 4 weeks in vivo, offering a suitable degradation rate matching the wound healing process.

The J‐TP demonstrated in this article represents an early stage of a hemostatic sealing agent that aims to simultaneously address the weak adhesive performance on bleeding tissues and the general but serious postoperative adhesion issues of the existing bioadhesives. Though further in‐depth investigations, such as the long‐term toxicity of the materials, ultimate clearance of the degradation by‐products, scale‐up animal study, and applicability on other tissues/organs will be required. The comprehensive in vitro, ex vivo, and in vivo tissue adhesion ability, antifouling, biodegradability, and hemostatic evaluations systematically validated the advantages of this *Janus* adhesive in inhibiting post‐operation tissue adhesion, stopping the bleeding of injured tissues, and promoting the healing of injured tissues, superior to that commercially available, widely clinic used hemostatic cyanoacrylate glue. Moreover, the design of J‐TP that was demonstrated here provides valuable insights for the future development of multifunctional bioadhesives for clinic applications.

## 4 Experimental Section

### Materials

Gelatin (type A bloom 300 from porcine skin) was purchased from Sigma‐Aldrich. Gelatin methacrylate (GelMA) with 60% substitution was prepared from gelatin according to published protocol.^[^ ^44^ ^]^ Acrylic acid (AA, 99%), N‐(methacryloxy)succinimide (NHSMA, 98%), polylactic acid (PLA, Mw ≈60 000), and 2,2‐azobis(2‐methylpropionitrile) (AIBN, 99%) were purchased from Aladdin. Irgacure 2959 and *α*‐ketoglutaric acid were obtained from Adamas. Methacryloyloxymethyl phosphonic acid (MMPA) was prepared according to the previous work.^[^ ^45^ ^]^ The photoreactive copolymer of poly(MPC‐*co*‐pMBP, 80:20) was supplied by Mono‐poly New Materials Scientific (Nanjing) Co., Ltd. Cyanoacrylate glue was supplied by Dragon Heart Medical Devices Co., ltd. All solvents were purchased from Sinopharm Chemical Reagent Co., Ltd. Phosphate‐buffered saline (PBS) and L929 cells were purchased from Adamas. Cell culture medium and cell counting kit‐8 (CCK8) were purchased from Beyotime Biotechnology. 6‐week‐old female Sprague–Dawley (SD) rats were purchased from Beijing Vital River Laboratory Animal Technology Co., Ltd., and related experiments were approved by the Ethics Committee of Nanjing Normal University and performed in accordance with the Institutional Animal Guidelines (IACUC‐1604001).

### Preparation of GelMA

GelMA was prepared through the acylation reaction between gelatin and methacrylic anhydride according to the published protocol.^[^ ^44^ ^]^ Briefly, 5 g of gelatin type A was dissolved in 50 mL PBS at 50 °C. Then, 8 mL of methacrylic anhydride was added to the gelatin solution dropwise, and the acylation reaction was performed under 50 °C for 3 h. The mixture was dialyzed in deionized water at 45 °C for 6 days through a dialysis membrane (Viskase, 14 kDa) and lyophilized for 3 days.

### Preparation of Poly(MMPA‐co‐NHSMA)

Poly(MMPA‐*co*‐pNHSMA) copolymers were prepared through photo‐polymerization of MMPA and NHSMA monomers under UV light irradiation. Typically, MMPA (7.2 mmol), NHSMA (0.8 mmol), and Irgacure 2959 (0.12 mmol) were dissolved in 4 mL of *N, N*‐dimethylformamide (DMF) and then placed in the UV chamber (365 nm, 10 W power) for 1 h. After photo‐polymerization, the mixture was precipitated in acetone and dried in vacuum, yielding a white powder (63% of yield). The chemical structure of the pure copolymer was analyzed by ^1^H NMR. The final poly(MMPA‐*co*‐pNHSMA) copolymer here refers to pMN in the following text.

### Preparation of the *Janus* Tissue Patch

The J‐TP contained three layers: a dry hydrogel adhesive bottom layer, a thin PLA plate middle layer, and a zwitterionic top layer (on one side of the PLA plate). To prepare the antifouling brush on a PLA plate, an ethanol solution of poly(MPC‐*co*‐pMBP, 80:20) (5 mg mL^−1^) was deposited on the surface of a glass slide in a customized mold. After evaporation of ethanol, a dichloromethane solution of PLA (10 mg mL^−1^) was placed on the poly(MPC‐*co*‐pMBP, 80:20) layer deposited on glass slide. After the evaporation of the solvent under room temperature, a transparent solid (thickness of about 0.01 mm) was formed in the mold. Then, UV irradiation (365 nm for 30 s with a density of 220 mW cm^−2^) was applied to the mold to obtain the zwitterionic polymers grafted PLA plate (PLA‐PMPC). The surface elemental compositions of the PLA‐PMPC plate were monitored by X‐ray photoelectron spectroscopy (XPS, ThermoFisher). The wettability of PLA, PLA‐PMPC with/without UV irradiation (PLA‐PMPC‐UV(+), PLA‐PMPC‐UV(−)) surface after ultrasonic cleaning in ethanol/H2O (v/v = 1:1) for 5 min was evaluated by water contact angle with a contact angle meter (KRUSS). The surface topography of the PLA‐PMPC membrane was imaged via scanning electron microscope (SEM, JEOL) after spraying with gold.

To prepare the J‐TP, AA (26 w/v%), pMN copolymers (4 w/v%), NHSMA (1 w/v%), gelatin (10 w/v%), GelMA (0.25 w/v%), and *α*‐ketoglutaric acid (0.3 w/v%) were dissolved in deionized water. The mixture was deposited onto the plasma‐treated PLA‐PMPC plate (treated by air plasma for 20 s with a voltage of 330 V and electricity of 101 mA before peeled off from the glass slide) and then cured under UV light (365 nm, 100 mW) for 30 min. After drying in air, the resulting *Janus* tissue patch was sealed and stored at −20 °C before use. The dry hydrogel‐based adhesive patch without PLA‐PMPC plate (referred to as TP) was obtained by curing the precursor solution in the mold under UV light and drying it in air. The composition of TP was analyzed by XPS. Hydrogel‐based adhesives without MMPA components were used as a control, which was prepared by replacing pMN polymers with an equal amount of AA.

### Biocompatibility Assay

The cytocompatibility of J‐TP was evaluated with mouse fibroblast cell line L929 according to the published protocol.^[^ ^16^ ^]^ Typically, the extraction medium was prepared by incubating 20 mg of dried J‐TP in 1 mL of high glucose DMEM medium supplemented with 10% FBS (fetal bovine serum) at 37 °C for 24 h. L929 cells (1 × 10^4^ cells mL^−1^) were pre‐seeded in 96‐well plates and cultured for 24 h. Then, the medium in each well was replaced with the J‐TP extraction medium and cultured at 37 °C for 24 h. Pristine DMEM with 10% FBS was used as a control (*n* = 6). Cell viability was quantitatively analyzed by CCK‐8 cell viability kit. Live/dead cells were stained with the calcein/PI cell viability/cytotoxicity assay kit (produced by Beyotime) and imaged by a fluorescence microscope (Nikon, Ti‐S).

Hemolysis assay was an easy and trustworthy approach to evaluating the blood compatibility of materials. The hemocompatibility of J‐TP was evaluated by the hemolysis ratio of J‐TP according to the published protocol.^[^ ^46^ ^]^ Red blood cells (RBC) were separated from the fresh blood of rats and suspended in PBS (2 v/v%). J‐TP discs (8 mm in diameter, 0.15 mm in thickness) were added into centrifuges tubes containing 3 mL of diluted RBC solution. The tubes were incubated for 1 h at 37 °C, centrifuged for 5 min at 3000 rpm, and the supernatant was collected. RBC in PBS and RBC in 2 v/v% Trition × 100 were set as the negtive control group and the positive control group, respectively. The absorbance of the supernatant was measured with a UV spectrophotometer at 540 nm and the hemolysis ratio was calculated according to the equation: hemolysis ratio (%) = [(A_E_ − A_P_)/(A_T_ − A_P_)] × 100%, where A_E_, A_T_, and A_P_ were the absorbance of J‐TP, positive control group, and negative control group, respectively.

### In Vitro Biodegradation Test

The in vitro biodegradation of J‐TP was evaluated by an enzymatic degradation process according to a published protocol.^[^ ^47^ ^]^ The enzymatic degradation medium was prepared by adding 2.5 mg collagenase into 100 mL DPBS (with calcium and magnesium) with an extra 0.01% (w/v) sodium azide added to prevent the growth of microorganisms. The J‐TP (about 50 mg) were immersed in 10 mL of freshly prepared degradation medium and placed at 37 °C under shaking (60 rpm) with regular changes of the degradation medium (every three days). The J‐TP incubated in DPBS without collagenase was set as the control group (*n* = 3). At each time interval, the samples were taken out from the incubation medium, washed with deionized water, frozen‐dried, and weighed. The degradation profile of J‐TP was determined by the percentage ratio between the real‐time weight and the original weight.

### Mechanical Test

The mechanical property of swollen J‐TP and TP was evaluated by a dynamic mechanical analysis (DMA) machine (Jinan Metex Test Technology Co., LTD, CMT6203). Before measurement, the dry TP and J‐TP adhesives were soaked in deionized water for several days to get swollen equilibrium. The swollen TPs and J‐TPs (dimension of 1.5 × 3 cm, thickness of 250 µm in a fully swollen state) were fixed onto the DMA machine and applied at a constant rate of 50 mm min^−1^. A mechanical sensor (100 N load cell) was used to monitor the dynamic force and the corresponding tensile strength was automatically calculated from the stress‐strain curves.

The adhesive performance of J‐TP was tested on the DMA machine. Prior to the test, tissue samples were soaked in PBS for 10 min, and the J‐TP adhesive was applied to the tissue surface at 37 °C with a gentle pressing of 5 kPa for 5 min. Interfacial adhesive strength between tissue samples (porcine skin, liver, heart, and stomach) and dry J‐TP (dimension of 2 × 2 cm, thickness of 250 µm) was measured at a constant tensile rate of 50 mm min^−1^. For the cohesive tests, samples were fixed to the aluminum holders through super glue (DELI). The force during the separation of J‐TP adhesive and tissues was monitored by a mechanical sensor (2 kN). The tensile strength was calculated by dividing the maximum force by the adhesive area. For the long‐term tensile strength test, J‐TP was applied to tissues at 37 °C with a gentle pressing of 5 kPa for 5 min, and the adhesive joints were sealed in plastic bags in wet environments for 24 h before testing. Three specimens per group were tested.

Bursting pressure tests were adapted according to a published protocol.^[^ ^48^ ^]^ Typically, porcine skin tissues with a 2‐mm‐diameter penetrating defect in the middle were fixed onto a silicone tube by super glue. The silicone tubing was linked to a syringe and a pressure gauge through a three‐way tube. The J‐TP (diameter of 12 mm, thickness of 0.1 mm) was used to seal the defect, with gentle pressure (2.85 kPa) applied on the J‐TP surface for 5 min. Then, the assembled device was filled with PBS through the syringe. Upon the gradual injection of PBS into the silicone tubing, the pressure inside the assembled device was gradually increased as monitored. A pressure gauge (rangeability of 100 kPa, Shanghai Tianchuan) was used to record the pressure in the tube. The maximum pressure before the leak of PBS from the damaged J‐TP was recorded as the bursting pressure. TP without a PLA backing plate was used as the control group.

### Clotting Assay

The hemostatic performance of pMN polymers and J‐TP were evaluated by the clotting time assay according to a published protocol.^[^ ^49^ ^]^ For the clotting assay of pMN polymers, 20 µL of phosphonic polymers in saline solution (0.05 g mL^−1^) was deposited in each well on a 96‐well plate. Then, 50 µL of recalcified rat blood (prepared by adding 0.1 M CaCl_2_ into the citrated blood) was added to each well. At predetermined time points (1, 2, 3, 4, 5, 6, and 7 min), the mixture in each well was washed with saline solution (0.9 wt% NaCl) until the washing liquid became clear, and the residual blood clot in the well was imaged by digital camera. The saline solution and the saline solution containing acrylic polymers (0.05 g mL^−1^) were set as the negative and positive controls, respectively.

The hemostatic ability of J‐TP was evaluated by a blood contact test. Briefly, recalcified rat blood (30 µL) was dropped on the surface of J‐TP, J‐TP without pMN polymers, and tissue culture plate. At predetermined time points (5 and 7 min), all samples were washed with 2 mL of saline solution. The washed samples were imaged by digital camera while the washing solutions were centrifuged at 1500 rpm for 10 min to collect the red blood cells. Then 2 mL of deionized water was added to lyse the red cells. The extracting solutions were imaged and the optical density (O.D.) of the solutions was recorded on a microplate at 450 nm to quantitatively analyze the amounts of matrix from lysed red cells.

### In Vitro Antifouling Test of the Zwitterionic Coating on J‐TP

All samples were sterilized by UV irradiation for 30 min before the test. For the antibacterial test, the PLA membranes with/without poly(MPC‐*co*‐pMBP) coating were co‐cultured with bacterial suspension (3 × 10^6^ CFU mL^−1^ in tryptone soy broth) under shaking at 100 rpm for 12 h. After rinsing with sterilized PBS, the bacteria on surfaces were stained with SYTO 9 in a LIVE/DEAD BacLight Bacterial Viability Kit (Thermo Fisher L7012) and imaged using a fluorescence microscope. For the cell adhesion test, the pristine PLA and PLA‐PMPC membranes (the same size as the well of a 24‐well plate) were pre‐immersed in the DMEM medium with high glucose containing 10% FBS for 12 h. After rinsedwith fresh DMEM medium, samples were placed at the bottom of a 24‐well plate. Then 1 mL of L929 fibroblast cell suspension (5 × 10^4^ cells mL^−1^) was dropped on the surface of each sample and incubated at 37 °C under a 5% CO_2_ atmosphere for 24 h. After rinsing with fresh DMEM medium and PBS, the attached cells on the sample surfaces were stained with a calcein/PI cell viability/cytotoxicity assay kit (Beyotime) and imaged by a fluorescence microscope.

### In Vivo Biodegradability Test

A subcutaneous embedding model on rats was used to evaluate the in vivo biodegradability of J‐TP according to the reported study.^[^ ^16^ ^]^ All samples (rectangle, 0.5 × 1 cm, thickness of 0.1 mm) were sterilized under UV irradiation for 3 h before use. After the anesthesia of a rat, a 2 cm skin incision was created on the rat's back and blunt dissection was performed from the incision to create enough space for J‐TP implantation. After the implantation of J‐TP, the incision was closed by using non‐absorbable 4‐0 sutures. After 2 and 4 weeks of the implantation, subcutaneous regions around the previous incision were harvested and fixed in 4% paraformaldehyde for 12 h for the following histological analyses.

### In Vivo Tissue Adhesion Test

A subcutaneous embedding model on rats was used to evaluate in vivo anti‐tissue adhesion property of the top layer of J‐TP according to a published protocol.^[^ ^31^ ^]^ All samples (rectangle, 0.5 × 1.5 cm) were sterilized under UV irradiation for 3 h before use. After anesthesia, a 3 cm skin incision was created on both the left and right sides of the rat's back. Then TP and J‐TP were implanted subcutaneously. The wound was sutured using non‐absorbable sutures. On day 7, tissues/implant complexes were harvested and stained with hematoxylin and eosin (H&E) for histological analysis on a microscope (Nikon, Japan).

### In Vivo Hemostasis and Wound Closure Evaluation

The in vivo hemostasis performance of the J‐TP was tested on rat liver according to the published protocol.^[^ ^40^ ^]^ 6‐week‐old female Sprague–Dawley rats (SPF‐level experiment animals) were raised in cages and randomly divided into three groups. After anesthesia, the abdominal skin of rat was cut to expose the liver. A liver bleeding model was created by puncturing the liver with a needle (puncture depth of 4 mm, scratch of 5 mm), while a piece of filter paper was placed beneath the bleeding liver. Then the J‐TP (diameter of 14 mm, thickness of 0.1 mm) was immediately applied on the surface of the bleeding site and pressed for 10 s to seal the wound. After 5 min, the filter paper with absorbed blood was weighed, and the blood loss was determined by the increased weights of the filter papers. The untreated wound and the wound treated with commercial cyanoacrylate glue were used as negative control and positive control, respectively. After suturing the skin, the rats were kept in cages for another 5 days. The injured livers were harvested to observe the histopathologic changes with H&E staining.

## Conflict of Interest

The authors declare no conflict of interest.

## Supporting information

Supporting Information

Supplemental Movie 1

Supplemental Movie 2

Supplemental Movie 3

Supplemental Movie 4

Supplemental Movie 5

Supplemental Movie 6

Supplemental Movie 7

## Data Availability

The data that support the findings of this study are available from the corresponding author upon reasonable request.
