# Supplementary material for: An Adhesive/Anti‐Adhesive Janus Tissue Patch for Efficient Closure of Bleeding Tissue with Inhibited Postoperative Adhesion
Source: Adv Sci (Weinh). 2023 May 12;10(21):2301427. doi: 10.1002/advs.202301427 (PMC10375199; doi:10.1002/advs.202301427)
Supplement: Supplementary file 1 — Supporting Information [file ADVS-10-2301427-s001.pdf]

ADVANCED SCIENCE
Open Access

Supporting Information

for *Adv. Sci.*, DOI 10.1002/advs.202301427

An Adhesive/Anti-Adhesive Janus Tissue Patch for Efficient Closure of Bleeding Tissue with Inhibited Postoperative Adhesion

Wan Peng, Cheng Liu, Youjin Lai, Yanting Wang, Pingsheng Liu and Jian Shen**

Supporting Information

An adhesive/anti-adhesive Janus tissue patch for efficient closure of bleeding tissue with inhibited postoperative adhesion

Wan Peng, Cheng Liu, Youjin Lai, Yanting Wang, Pingsheng Liu, Jian Shen, **

W. Peng, Y. J. Lai, Y. T. Wang, Prof. P. S. Liu, Prof. J. Shen
Jiangsu Collaborative Innovation Center of Biomedical Functional Materials, Jiangsu Key Laboratory of Bio-functional Materials, School of Chemistry and Materials Science, Nanjing Normal University, Nanjing 210023, P. R. China
E-mail: liups@njnu.edu.cn
C. Liu
The Affiliated Drum Tower Hospital of Nanjing University Medical School, Nanjing 210093, P. R. China
Prof. J. Shen
Jiangsu Engineering Research Center of Interfacial Chemistry, Nanjing University, Nanjing 210093, P. R. China
E-mail: shenjian@nju.edu.cn

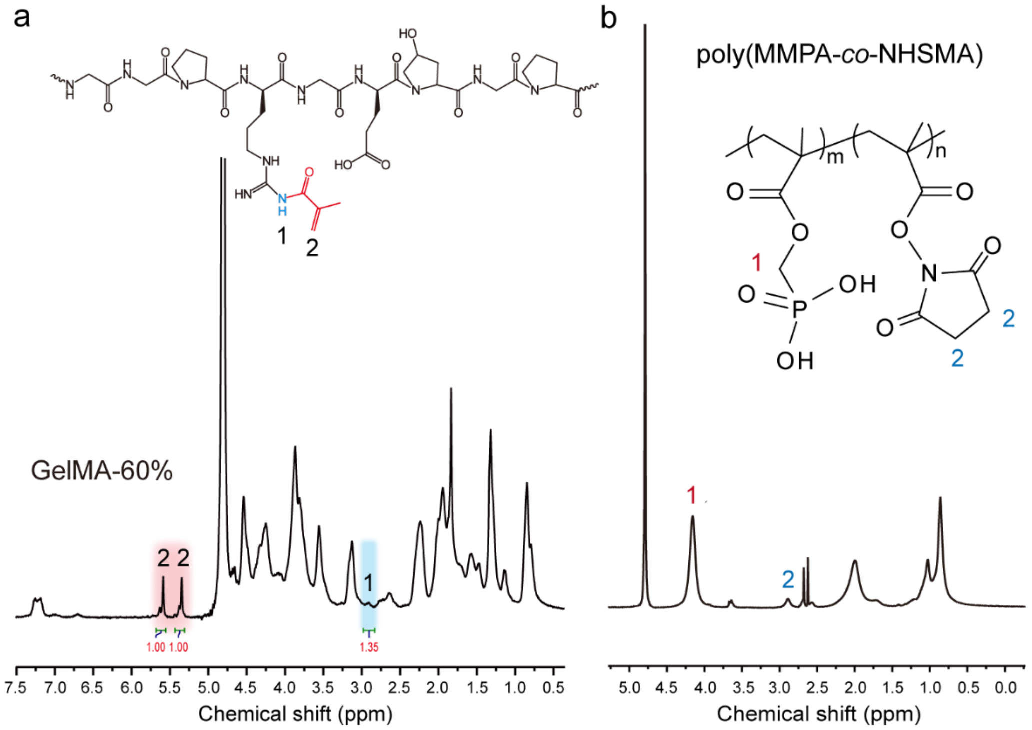

Figure S1. ^{1}H NMR spectra of gelatin methacrylate (a) with 60% substitution and (b) poly(MMPA-*co*-pNHSMA) copolymers (n_{MMPA} : n_{NHSMA}=90:10). The degree of

methacrylation of gelatin was calculated from the integration of the peak areas in the ^{1}H NMR spectra (Degree of methacrylation=$I_1/(I_1+I_2)\times 100\%$, I is the integration of relative peak areas).

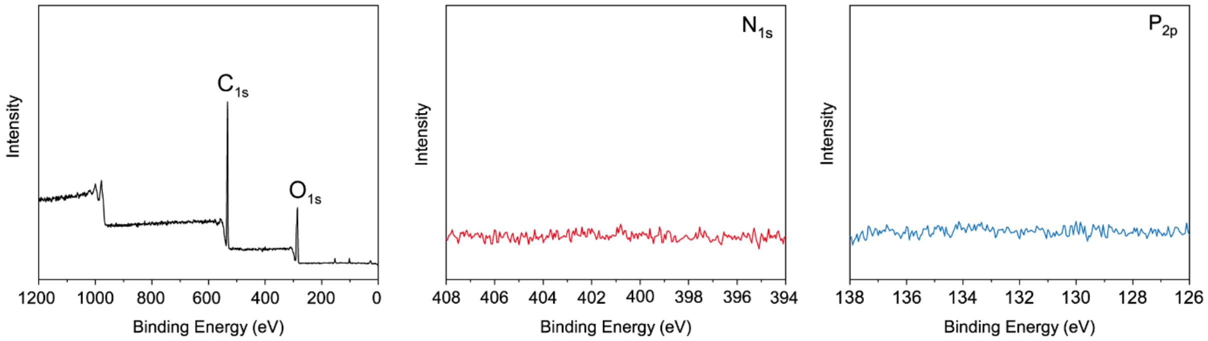

Figure S2. Without UV irradiation, XPS survey scan, N_{1s} and P_{2p} high-resolution scan spectra of ultrasonic cleaning PLA layer deposited on p(MPC-co-MBP) copolymers.

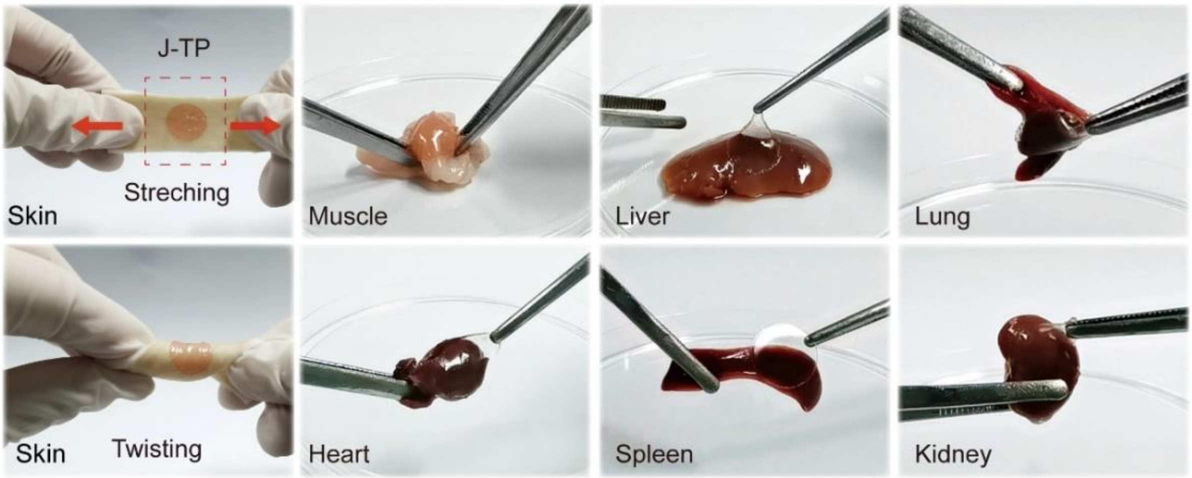

Figure S3. Images of the J-TP adhered to diverse tissues within 15 s (porcine skins and muscles, rat's heart, liver, lung, spleen and kidney).

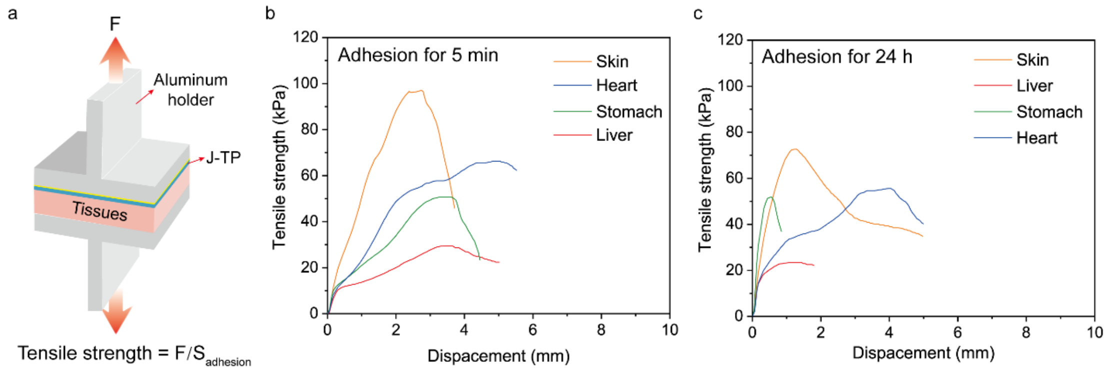

Figure S4. Representative curves for tensile tests of various tissues adhered by the J-TP. (a) Schematic illustration of tensile tests of tissues adhered by the J-TP. Tensile strength *vs.* displacement curves for tensile tests of various tissues adhered by the J-TP for (b) 5 min, and (c) 24 h.

Supplementary Movie S1.

Adhesion for porcine stomach: Prior to the application of J-TP, obvious leakage of PBS was observed at the penetrating defect (7-mm) on porcine stomach. After applying the transparent J-TP (diamater of 25 mm, thickness of 0.1 mm) at the defect site for 15 seconds, the leakage was stopped.

Supplementary Movie S2.

Adhesion for porcine heart: After the application of J-TP (diamater of 25 mm, thickness of 0.1 mm) on the surface of porcine heart with gentle press for 10 seconds, obvious adhesion occurred between J-TP and porcine heart.

Supplementary Movie S3.

Bursting pressure - without PLA: The tissue patch without PLA backing (diameter of 12 mm) was applied to seal the 2-mm-diameter penetrating defect in porcine skin (with gentle pressure applied on the J-TP surface for 5 min). The maximum pressure before the leak of PBS from the damaged J-TP was recorded as the bursting pressure.

Supplementary Movie S4.

Bursting pressure - with PLA: The tissue patch with PLA backing was applied to seal the 2-mm-diameter penetrating defect in porcine skin with the same operation in movie S3**.**

Supplementary Movie S5.

Liver bleeding - none treatment: A piece of filter paper was placed beneath the bleeding liver, and the rat liver was punctured with a needle (puncture depth of 4 mm, scratch of 5 mm). Without any hemostasis treatment, the blood loss of liver was recorded by the increased weights of filter paper.

Supplementary Movie S6.

Liver bleeding - cyanoacrylate glue: A bleeding liver model was created with the same operation in movie S5. About 50 μL of commercial cyanoacrylate glue was immediately applied on the bleeding site to stop the bleeding. The blood loss of liver was recorded by the increased weights of filter paper.

Supplementary Movie S7.

Liver bleeding - J-TP: A bleeding liver model was created with the same operation in movie S5. The J-TP (diameter of 14 mm) was immediately applied on the surface of bleeding site and pressed for 10 seconds to seal the wound. The blood loss of liver was recorded by the increased weights of filter paper.
